# Ethanol-induced lymphatic endothelial cell permeability via MAP-kinase regulation 

**DOI:** 10.1152/ajpcell.00039.2021

**Published:** 2021-04-28

**Authors:** Matthew Herrera, Patricia Molina, Flavia M. Souza-Smith

**Affiliations:** Department of Physiology, Alcohol and Drug Abuse Center of Excellence, Louisiana State University Health Sciences Center, New Orleans, Louisiana

**Keywords:** alcohol, endothelial cell, lymphatic, MAPK, tight junction

## Abstract

Chronic alcohol alters the immune system enhancing the susceptibility to inflammation, bacterial, and viral infections in alcohol users. We have shown that alcohol causes increased permeability of mesenteric lymphatic vessels in alcohol-fed rats. The mechanisms of alcohol-induced lymphatic leakage are unknown. Endothelial cell monolayer permeability is controlled by junctional proteins complexes called tight junctions (TJ) and adherens junctions (AJ), and each can be regulated by MAPK activation. We hypothesize that ethanol induces lymphatic endothelial cell (LEC) permeability via disruption of LEC TJ through MAPK activation. An in vitro model of rat LECs was used. Ethanol-supplemented medium was added at concentrations of 0, 25, and 50 mM to confluent cells. Resistance-based barrier function, transwell permeability, cell viability, TJ, AJ, and MAPK protein activity, TJ and AJ gene expressions, and the role of p38 MAPK in barrier function regulation were measured. Ethanol increased the permeability of LECs compared to controls that was not associated with decreased cell viability. LECs treated with 50 mM ethanol showed an increase in phosphorylated levels of p38. No significant changes in TJ and AJ gene or protein expressions were observed after ethanol treatment. p38 inhibition prevented ethanol-induced increases in permeability. These findings suggest that p38 may play a role in the regulation of ethanol-induced LEC permeability, but altered permeability may not be associated with decreased TJ or AJ protein expression. Further investigation into junctional protein localization is needed to better understand the effects of ethanol on lymphatic endothelial cell-to-cell contacts and hyperpermeability.

## INTRODUCTION

Excessive alcohol consumption is associated with alterations in inflammatory and immune responses and can cause increased susceptibility to infection (1). Alcohol can also affect the barrier integrity of various tissues. For example, the function of the intestinal barrier can be impaired in people with alcohol use disorder, causing increased permeability of the gut to macromolecules and contributing to inflammatory processes seen in alcohol liver disease (2–4).

As a conduit for the immune system, the lymphatic system (LS) is believed to play a key role in these changes observed during alcohol consumption. The LS is a network of vessels throughout the entire body that plays an important role in tissue fluid pressure regulation, immune surveillance, and absorption of dietary fats in the intestine (5–8). Although it has not been studied extensively until recent decades, the lymphatic system is known to contribute to numerous diseases, such as lymphedema, cancer metastasis, and several inflammatory disorders (5, 9, 11–13). The circulation of lymph fluid throughout the LS allows for proper immune dialogue between tissues and lymph nodes (14), which assist to generate a specialized microenvironment for the meeting of migratory immune cells, such as lymphocytes and antigen presenting cells (APCs) (15, 16).

Compromised lymphatic integrity, characterized by hyperpermeable lymphatic vessels, decreased pumping capacity, and decreased migration of immune cells through the LS, can lead to leakage of lymph into nearby surrounding tissues, leading to inflammation and decreased trafficking of immune cells through the lymphatic system (6, 17–22). We have previously shown that alcohol administered acutely, in a binge, and chronically in the diet increases lymphatic vessel permeability (23–25). Our data show that alcohol-induced lymphatic hyperpermeability disrupts immune cell trafficking and may contribute to the development of metabolic dysregulation. However, the mechanisms involved in this alcohol-induced lymphatic vessel hyperpermeability is not yet known.

In vitro, ethanol is known to increase permeability of numerous endothelial cell monolayers via disruption of junctional protein integrity through activation of various different mechanisms (26–30). The lymphatic endothelial cells (LECs) that make up the collecting lymphatic vessels of the LS contain transmembrane junctional protein complexes that are involved in regulating barrier function of the endothelia. These complexes, called tight junctions (TJ) and adherens junctions (AJ), are important for the formation and maintenance of barrier function of endothelial cells (31–33). Both junctional complex sites of attachment between endothelial cells operate as signaling structures that communicate and regulate various cell properties and functions, such as position, growth, and vascular homeostasis (34, 35). Any change in junctional integrity, organization, protein expression, or function may have complex consequences that could compromise endothelial barrier function (36–43). For example, deletion of a single copy of a gene crucial for the formation of lymphatic vasculature causes structural changes in lymphatic vessels that lead to leakage of lymph from the LS into surrounding tissues (44). In addition, factors that disrupt barrier function in vitro can lead to alterations in cell-cell junction protein distribution resulting in increased permeability (45).

TJs are responsible for the initiation and maintenance of intercellular adhesion, and their expression is known to be disrupted during states of increased permeability (36, 46, 47). Previous studies have shown that alcohol-induced vascular permeability is associated with increased activation of the mitogen-activated protein kinase (MAPK) pathway (30). Activation of the MAPK signaling pathway can lead to modulation of TJ paracellular transport via up- or down-regulation of TJ protein expression, altering the protein composition within TJ complexes and causing increases or decreases in endothelial cell permeability (30, 48–50). Additional supporting evidence for the role of MAPK activation comes from studies that show inhibition of MAPK prevents increases in permeability (26, 51, 52). Although MAPK activation has been implicated as a mechanism involved in the regulation of barrier function of vascular endothelial cells (30, 49), its specific role in the regulation of lymphatic endothelial cell barrier function is not fully understood. Although the mechanism involved in alcohol-induced leakage of various tissue types have been widely studied, the mechanisms underlying alcohol-induced lymphatic leakage are not yet identified and are the target of our present studies.

We hypothesized that ethanol activates LECs MAPK signaling, leading to decreased TJ and/or AJ protein expression and subsequently disrupting the lymphatic endothelial barrier. This decreased barrier function would cause increased permeability of the lymphatic endothelial cell monolayer. Using Electrical Cell-Substrate Impedance Sensing (ECIS) and transwell permeability assays to measure changes in resistance and molecule migration, respectively, across the LEC monolayer, we demonstrated that ethanol caused a disruption of the endothelial barrier and enhanced migration of molecules across the LEC monolayer. This increased permeability was prevented by inhibition of p38 MAPK activity.

## METHODS

### LEC Culture

Sprague–Dawley rat primary dermal lymphatic endothelial cells, isolated from skin tissue of one-day-old neonatal rats from a pool of rats (sex unknown) were obtained from Cell Biologics (Cat. No. RN-6064L; Chicago, IL). Before seeding cells, plates were first incubated with gelatin-based solution (Cell Biologics; Cat. No. 6950; Chicago, IL) for 2 min. Cells were then seeded onto plates and cultured with complete rat endothelial cell medium (Cell Biologics; Cat. No. M1266 w/kit; Chicago, IL). LECs were used between passages 2 and 5. Cells were incubated at 37°C and 5% CO_2_ and grown to confluent monolayers before the initiation of experiments. For ethanol treatment experiments, ethanol-supplemented cell culture medium at concentrations of 0, 25, and 50 mM were used in all experiments described. Cells cultured in the absence of ethanol (0 mM) served as controls unless otherwise stated. Cells in the ethanol-treated groups were treated with ethanol-supplemented medium at 25 mM and 50 mM ethanol and cultured in incubators with an ethanol-water solution in the humidification tray maintained at 50 and 75 mM ethanol, respectively. This approach limits evaporation of and loss of ethanol in the culture media during experiments (53).

### Assessment of Transendothelial Resistance

LEC barrier function was measured by electrical resistance across the endothelial cell monolayer using Electrical Cell-Substrate Impedance Sensing (ECIS) 1600R system from Applied Biophysics (Applied Biophysics; Cat. No. 3502ZZMFG; Troy, NY). The protocol was followed as previously described (53–56). Briefly, eight-well ECIS disposable arrays (Applied Biophysics; Cat. No. 8W10E+; Troy, NY) containing electrodes embedded in the plates were first stabilized and precoated with a gelatin-based solution (Cell Biologics; Cat. No. 6950; Chicago, IL). Following stabilization pretreatment, 400 µL of medium containing approximately 2.5 × 10^5^ cells/mL was added to each well [*n* = 8 for all transendothelial resistance (TER) experiments]. During cell seeding, and all medium changes, medium warmed to 37°C was used to match the temperature of the incubator and to prevent any effects of temperature in the system. After cell seeding, transendothelial resistance was monitored as cells grew to confluence. Confluence in this system was defined to be the point at which the resistance plateaued and remained approximately constant for 12–24 h.

Once a confluent cell monolayer was reached, TER measurement was paused, and the normal culture medium was removed from all of the wells of the ECIS array. Fresh ethanol-supplemented or control medium was then added to each well. Because only one ECIS array could be used at a time to collect data, each array was time-matched for treatments in each respective treatment group. Ethanol-supplemented or control medium was added to each well and TER was continuously monitored for 24–48 h. TER of control and 25 mM ethanol treatments was measured for 24 h. TER of 50 mM ethanol treatments was measured for 48 h to observe recovery of TER back to baseline resistance. Cells cultured for 48 h received 24 h of 50 mM ethanol treatment followed by replacement of experimental culture medium with normal cell culture medium for an additional 24 h to observe TER recovery. Once measurements were finished, data were normalized to the baseline resistance that was measured before the addition of treatment medium. Based on the results from these ECIS studies done to measure TER, the appropriate time point for ethanol treatment was chosen and used in further protein analysis.

### Time Point Rationale for Characterization of LEC Dynamics

Previous studies with various endothelial cells types have explored the potential MAPK pathways involved in inducing cellular permeability and changes in TER. When various permeability-inducing agents, including ethanol, were studied, MAPK activation was observed between 20% and 100% of the time it took to reach the maximum change in resistance (11, 57–59). In these present studies using LECs and the TER technique, we found that the time it took for resistance to reach its maximum decrease was ∼10 h for 25 mM ethanol and ∼15 h for 50 mM ethanol. After considering the trends in MAPK activation seen in the previously described literature and the results presented here, it was decided that the appropriate time to measure MAPK activation was at 50% of the maximum change in resistance elicited by the highest dose of ethanol. Therefore, 7.5 h of ethanol treatment was used this time point represents 50% of the time it took for cells receiving 50 mM ethanol to reach the maximum change in resistance. Additionally, measurements of LEC barrier function and protein expression after 30 min of ethanol were performed to observe if the ethanol-induced effects on protein expression or barrier function seen at 7.5 h were also present at early time points of ethanol treatment.

### Transwell Permeability

A transwell permeability assay (Cell Biologics; Cat. No. CB6929; Chicago, IL) was used to further assess changes in LEC monolayer permeability (21, 60). Briefly, LECs were seeded onto 24-well cell culture transwell inserts and grown to confluence. Once cells were confluent, the bottom chambers received 1 mL of control or ethanol-supplemented culture medium. 300 µL of the same medium was added to the top chamber. 5 µL of HRP (44 kDa) was also added to the top chamber of the inserts. Following either 30 min or 7.5 h of ethanol treatment, a 20-µL aliquot of the medium from the bottom chamber placed into a well of a 96-well plate. 50 µL of the 3,3’,5,5’-tetramethylbenzidine (TMB) substrate solution provided in the assay kit was added to the aliquot and mixed for 2–3 min. 50 µL of the stop solution provided by the assay kit was then added to the mix, which stopped the reaction occurring between the HRP and TMB substrate and caused the mixture to turn a yellow color. The spectrum absorbance of this yellow mixture was then measured at 450nm using a spectrophotometer (BioRad; Hercules, CA). Data are presented as relative permeability normalized to control values. *N* = 4 for 30-min time point, and *n* = 8 for 7.5-h time point.

### MTT and LDH assays

The 3-(4,5-dimethylthiazol-2-yl)-2,5-diphenyltetrazolium bromide (MTT) assay (Thermo Fisher, No. V13154) was employed to determine the number of viable cells in culture after exposure to ethanol. Briefly, LECs were plated into 96-well plates and grown to confluence. Cells were then exposed to ethanol for indicated times. Following ethanol treatment, 10 µL of 12 mM MTT reagent was added into each well, and the plates were incubated at 37°C for 4 h. The cultures were then solubilized using SDS-HCl solution at 37°C for 4 h. Spectrophotometric absorbance was measured at 570 nm using a microplate reader (59, 61, 62). Data are expressed as relative cell viability. *N* = 6.

To measure potential ethanol-induced cytotoxicity of cells, the CyQUANT LDH Cytotoxicity Assay Kit (Thermo Fisher, No. C20300) was used. The protocol used for the LDH assay was provided by the manufacturer (63). Briefly, cells were plated in 96-well flat bottom plates and grown in the same manner as those used in the MTT assay. After experimental treatment, 50 µL of experimental culture medium were removed from each well and placed into a new 96-well plate. 50 microliters of LDH reaction mixture was added to these new plates and then plates were incubated for 30 min at room temperature. Following incubation, the stop reagent was added, and absorbance was measured at 490 and 680 nm to determine both experimental absorbance and background absorbance. In addition to the experimental treatments, maximum LDH release was measured by treating a separate group of cells with lysis buffer prior to treating them with reaction and stop reagents in similar manner to experimental groups. LDH release was calculated as a percentage of maximum LDH release and normalized to control values. *N* = 6.

### Western Blot Analysis

Western blots were performed to assess changes in TJ protein expression after exposure to ethanol. Briefly, following either 30 min or 7.5 h of ethanol exposure, cells were washed with PBS, harvested, and lysed with Tissue Protein Extraction reagent (Thermo Scientific, Cat. No. 78510; Waltham, MA). Approximately 25 µg of protein, as determined by the Pierce BCA protein assay kit (Thermo Fisher; Cat. No. 23225; Waltham, MA), were separated by SDS-PAGE (4-15% polyacrylamide gel) and transferred to polyvinylidene difluoride (PVDF) membrane (EMD Millipore; Cat. No. ISEQ00010; Burlington, MA). Membranes were blocked with either 5% BSA or nonfat milk and incubated with the primary antibodies at 4°C overnight in 2.5% BSA or nonfat milk. Primary antibodies and secondary antibodies used are listed in Table 1 and original blot pictures are shown in Supplemental Fig. S1 (https://figshare.com/s/ee8b8f15d2c51492ad05). For MAPK Western blot analysis, *n* = 10; for TJ and AJ Western blot analysis, *n* = 5–9.

**Table 1.** Primary and secondary antibodies used for Western blotting

	Dilution	Manufacturer (Product #)
*Primary antibody*		
P-p38	1:1,000	Cell Signaling (4511)
P38	1:1,000	Cell Signaling (8690)
P-p44/p42 (P-Erk1/2)	1:1,000	Cell Signaling (9106)
p44/p42 (P-Erk1/2)	1:2,000	Cell Signaling (9102)
P-SAPK/JNK	1:1,000	Cell Signaling (9251)
SAPK/JNK	1:1,000	Cell Signaling (9252)
P-β-catenin	1:1,000	Sigma Aldrich (4300630)
β-Catenin	1:1,000	Sigma Aldrich (06734)
P-p120-catenin	1:1,000	Sigma Aldrich (4504158)
p120-catenin	1:1,000	Sigma Aldrich (4500550)
GRB2	1:1,000	Cell Signaling (3972)
ZO-1	1:1,000	Thermo Fisher (40-2200)
Claudin-5	1:1,000	Thermo Fisher (35-2500)
Occludin	1:1,000	Thermo Fisher (71-500)
*Secondary antibody*		
Anti-mouse	1:10,000	Bio-Rad (170-6516)
Anti-rabbit	1:10,000	Cell Signaling (7074S)

GRB2, growth factor receptor bound protein 2; JNK, c-Jun N-terminal kinase; SAPK, stress-activated protein kinases; ZO, zonula occludens.

### Measurement of TJ and AJ Gene Expression using RT-qPCR

To measure gene expression of TJ and AJ proteins, RT-qPCR was performed for claudin-5, occludin, ZO-1, and VE-cadherin. RNA was extracted from LECs after treatment with ethanol for 7.5 h using the RNeasy Mini kit (Qiagen Sciences, Cat. No. 74104; Germantown, MD), as per the manufacturer’s instructions. cDNA was synthesized from 1 µg of total RNA using the Quantitect Reverse Transcriptase Kit (Qiagen Sciences; Germantown, MD). Primers (Table 2) were designed to expand exon-exon junctions and used at 500 nmoles (Integrated DNA Technologies; Coralville, IA). Final reactions were made to a total volume of 20 µL with the Quantitect SyBr Green PCR kit and DNase RNase-free water (Qiagen Science; Germantown, MD). All reactions were carried out in duplicate on the CFX96 system (BioRad; Hercules, CA*)* for qPCR detection. *N* = 6.

**Table 2.** qPCR primer sequences for TJ and AJ genes

Gene	Forward Primer	Reverse Primer
VE-Cadherin	GCA ACT TCA CCC TCA TCA A	CAG GTA GTG GAA CTT GGT ATG
Claudin-5	AGG CTC TTG TGA GGA CTT	GCA GTT TGG CTA CTT
ZO-1	CCA GTA CCC ACG AAG TTA TG	GGA AGG TAT CAG AGG AGG AA
Occludin	GCA GCA ACG ATA ACC TAG AG	GTC GTG TAG TCG GTT TCA TAG

AJ, adherens junction; TJ, tight junction; VE-cadherin, vascular endothelial cadherin; ZO, zonula occludens.

### Pharmacological Inhibition of p38 MAPK

Inhibition of p38 MAPK was performed to assess the role of p38 in ethanol-induced changes in permeability (64). For this purpose, cells were plated in eight-well ECIS arrays and allowed to grow to confluence in normal culture medium while TER was measured using the ECIS system as previously described. To inhibit p38 phosphorylation and activity, the p38 inhibitor SB203580 (Sigma Aldrich; Cat. No. 559389; St. Louis, MO) was used at 20 µM concentrations, as determined by dose-response tests done before using the inhibitor. Vehicle control was 1% DMSO diluted in 50 mM ethanol-supplemented medium, and the ethanol control was 50 mM ethanol-supplemented medium alone. Once cells reached confluence in the ECIS arrays, culture medium was removed from all wells of the array and pre-treated with the appropriate control or experimental inhibitor groups depicted in Fig. 6. In order to inhibit p38 activity, cells were first pre-incubated with inhibitor diluted in normal LEC culture medium for one hour. After one hour of this pre-treatment, medium was removed again and replaced with the appropriate inhibitor diluted in 50 mM ethanol. Cells were then incubated for 24 h. TER was continuously measured throughout the pre-treatment and ethanol treatment process. TER measured was compared between ethanol control, vehicle control, and inhibitor groups. For control and vehicle groups, *n* = 4; for inhibitor groups, *n* = 8.

### Statistical Analysis

Summarized data are indicated in the corresponding figure legends. Data are presented as mean ± SE. One-way ANOVA and Tukey’s post hoc test was used to compare changes in TER, protein expression, cell viability, and cell cytotoxicity. Student *t* test was used to compare changes in transwell permeability and gene expression. Differences in which *P* was less than 0.05 were considered statistically significant. Graphpad Prism 7 software was used in all analyses.

## Results

### Ethanol Disrupts LEC Barrier Integrity

**Figure 1.**
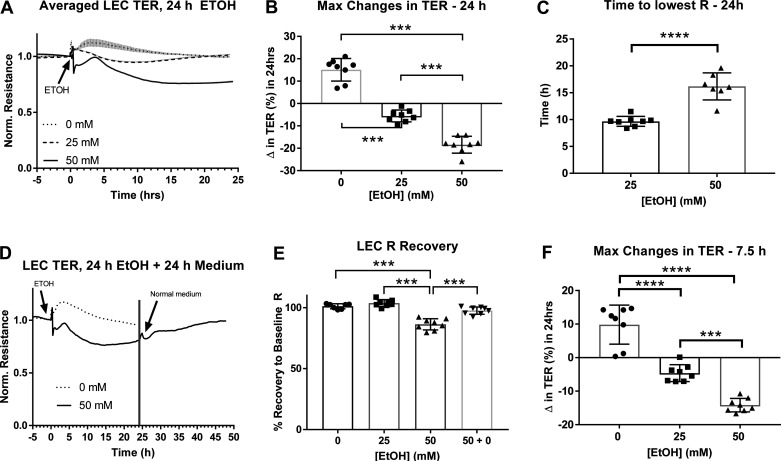
Changes in transendothelial resistance (TER) in lymphatic endothelial cells [LEC cultures after 24 h ethanol (EtOH) (0, 25, 50 mM)]. *A*: averaged tracings of each treatment group. *B*: maximum changes (Δ) in TER over the span of 24 h treatment with ethanol. *C*: average time to reach the lowest TER. *D*: tracing of TER recovery in 50 mM ethanol groups after removal of ethanol a resupplementation with normal culture medium for 24 more hours (48 h total). *E*: the percent recovery to baseline TER after 24 h treatment with ethanol. *F*: maximum changes (Δ) in TER over the span of 7.5-h treatment with ethanol. Y-axis values in (*A*) and (*D*) are resistance measurements normalized to time point before treatment began (time zero), X-axis represents the entire time course; Values in (*D*) are averages of values from (*A*); circle, square, and triangle plots represent the groups receiving 0, 25, or 50 mM ethanol for 24 h, respectively. Inverted triangle plots represent groups receiving 50 mM ethanol for 24 h followed by resupplementation with 0 mM normal culture medium for 24 more hours (48 h total). A total of three independent experiments were performed for Fig. 1 data. Values are mean ± SE; *n* = 8; (*B*) and (*E*)—one-way ANOVA, Tukey’s; (*C*)—*t* test; ***P < 0.001, ****P < 0.0001. R, resistance.

We tested the effects of ethanol on LEC barrier integrity. Over the span of 24 h, ethanol exposure caused a significant decrease in TER in a concentration-dependent manner (Fig. 1*A*). After addition of normal medium, the maximum change in TER was positive in LECs treated with 0 mM ethanol and negative in LECs treated with 25 and 50 mM ethanol, with a greater decrease caused by 50 mM ethanol compared to 25 mM ethanol (Fig. 1*B*). The same changes were found at 7.5 h, time point determined for molecular measurements (Fig. 1*F*). Cells treated with 50mM ethanol took longer to reach the lowest TER compared to cells treated with 25 mM ethanol (Fig. 1*C*). Over the span of 24 h, cells treated with 25 mM ethanol showed an almost complete recovery to baseline TER. Baseline TER is defined as the resistance that was measured before exposure to control or ethanol-supplemented medium. Cells treated with 50 mM did not fully recover to baseline TER, showing approximately 80% recovery (Fig. 1*E*). After 24 h of ethanol treatment, the ethanol medium of cells treated with 50 mM ethanol was removed and replaced with 0 mM culture medium. TER of this group alone was then monitored for another 24 h (48 h total). The results show recovery of TER close to baseline resistance (Fig. 1*D*).

**Figure 2.**
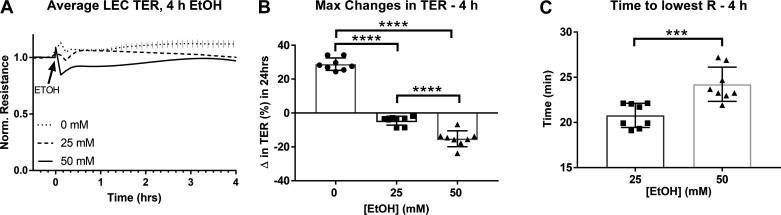
Changes in trans-endothelial resistance (TER) in lymphatic endothelial cells (LEC cultures after 4 h ethanol (EtOH) (0, 25, 50 mM). *A*: averaged tracings of each group. *B*: maximum changes (Δ) in TER over the span of 4 h treatment with ethanol. *C*: average time to reach the lowest TER. Y-axis values in (*A*) are resistance measurements normalized to time point before treatment began (time zero), X-axis represents the 4 h-time course. Circle, square, and triangle plots represent the groups receiving 0, 25, or 50 mM ethanol, respectively. A total of three independent experiments were performed for Fig. 2 data. Values are mean ± SE; *n* = 8; (*B*)—one-way ANOVA, Tukey’s; (*C*)—*t* test; ***P < 0.001, ****P < 0.0001. R, resistance.

Ethanol was also shown to cause earlier changes in TER than those measured over the span of 24 h (Fig. 2*A*). Within the first 4 h of treatment, 25 mM and 50 mM ethanol caused initial decreases in TER that were significantly different than the TER seen in control treatments (Fig. 2*B*). These changes occurred within the first 30 min of ethanol treatment, with greater and more sustained changes observed at higher concentrations of ethanol, like that seen over 24 h (Fig. 2*C*).

### The Effects of Ethanol on LEC Transwell Permeability

**Figure 3.**
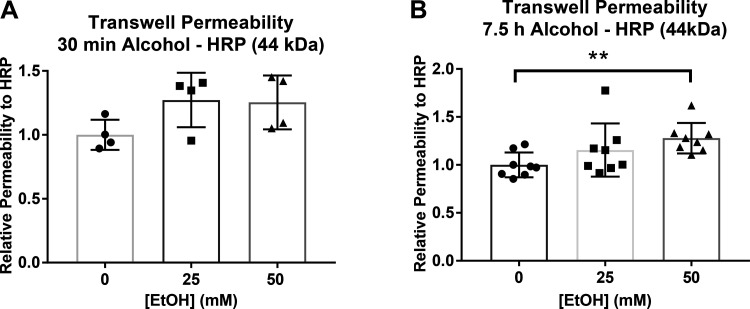
Relative permeability to horseradish peroxidase (HRP) of lymphatic endothelial cell (LEC) monolayer after treatment with ethanol (0, 25, 50 mM). *A*: transwell permeability to HRP at 30 min and (*B*) 7.5 h of ethanol (0, 25, 50 mM) relative to controls. Circle, square, and triangle plots represent the groups receiving 0, 25, or 50 mM ethanol. A total of three independent experiments were performed for Fig. 3 data. Values are mean ± SE; *n* = 4 for 30 min and *n* = 8 for 7.5 h; *t* test, **P < 0.01. EtOH, ethanol.

Although measurements of TER are an often-used technique to measure changes in permeability, we also assessed changes in permeability using transwell permeability assays. After treatment with ethanol for 30 min, LECs showed slight increases (*P* = 0.08) in relative permeability to HRP molecules as measured by the HRP transwell permeability assay (Fig. 3*A*). Treatment with 50 mM ethanol for 7.5 h significantly increased relative permeability to HRP compared to time-matched controls. LECs treated with 25 mM ethanol showed a modest but not statistically significant increase in permeability (Fig. 3*B*).

### The Effects of Ethanol on Cell Health

**Figure 4.**
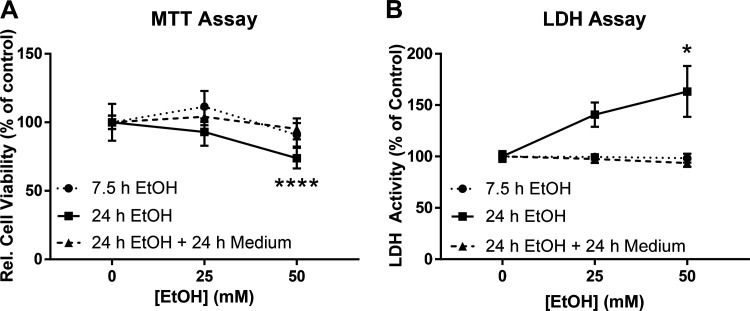
Relative cell viability and cell toxicity of LECs after treatment with ethanol (0, 25, 50 mM). *A*: cell viability after ethanol treatment measured by MTT assay. *B*: cell toxicity after ethanol treatment measured by LDH assay. In both graphs, the group representation is 7.5 h (circle) and 24 h (square) of treatment with ethanol. Triangle represents the 24 h ethanol + 24 h normal LEC culture medium (48 h total) group. A total of two independent experiments were performed for Fig. 4 data. Values are mean ± SE; *n* = 6; one-way ANOVA; *P < 0.05, ****P < 0.0001 compared to control 0 within group. EtOH, ethanol; LDH, lactate dehydrogenase; LEC, lymphatic endothelial cell; MTT, 3-(4,5-dimethylthiazol-2-yl)-2,5-diphenyltetrazolium bromide.

The MTT assay was used to assess changes in cell viability after exposure to ethanol. After 7.5 h of ethanol treatment, there were no significant changes in cell viability between groups. However, after 24 h of ethanol treatment, the 50 mM ethanol group showed a significant decrease in relative cell viability compared to time-matched controls. Cells that were treated with ethanol for 24 h, then washed and treated with normal LEC culture medium for another 24 h (48 h total) showed no significant differences in cell viability between groups (Fig. 4*A*). In contrast, average cell viability measures at 24 and 48 h were significantly increased as compared to the means obtained at 7.5 h.

In addition to measurements of relative cell viability and changes in confluence, the amount of cytotoxicity caused by exposure to ethanol was measured by the LDH assay. There were no significant differences in cytotoxicity after treatment with ethanol for 7.5 h. Only exposure to ethanol 50 mM for 24 h resulted in a significant increase in LDH release compared to time-matched controls. Treatment of cells with ethanol for 24 h followed by removal of ethanol and normal culture media supplementation for 24 more hours (48 h total) did not show significant differences in LDH release as compared to time-matched control cultures (Fig. 4*B*).

### The Effects of Ethanol on MAPK Activity

**Figure 5.**
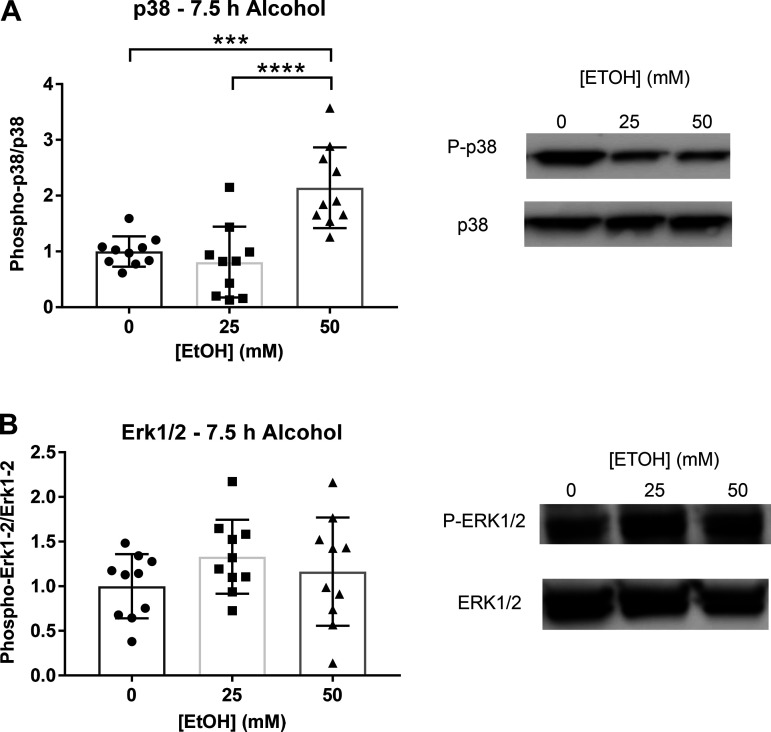
MAPK phosphorylation after either 7.5 h or 30 min of LEC treatment with ethanol (0, 25, 50 mM). *A*: p38 phosphorylation after treatment with ethanol (0, 25, 50 mM). *B*: Erk1/2 phosphorylation observed after 7.5 h ethanol. JNK MAPK could not be detected. Y-axis represent changes in phosphorylated protein/total protein. X-axis represents different concentrations of ethanol. Circle, square, and triangle plots represent the groups receiving 0, 25, or 50 mM ethanol, respectively. A total of three independent experiments were performed for Fig. 5 data. Values are mean ± SE, *n* = 10; one-way ANOVA, Tukey’s; ***P < 0.001, ****P < 0.0001. EtOH, ethanol; JNK, c-Jun N-terminal kinase; LEC, lymphatic endothelial cell.

We used Western blotting to test the effects of ethanol on MAPK activation. Cells were cultured with ethanol supplemented medium at 0, 25, and 50 mM concentrations to match the concentrations used during measurements of LEC permeability previously described, and MAPK activity was measured via Western blotting (WB). Cells treated with 50 mM ethanol for 7.5 h showed a significant increase in levels of phosphorylated p38 MAPK normalized to total protein (Fig. 5*A*). However, there were no significant changes in phosphorylated ERK1/2 between groups (Fig. 5*B*). Expression of c-Jun N-terminal kinase (JNK) could not be detected by WB. After 30 min of ethanol treatment, there were no significant changes in p38 phosphorylation (data not pictured).

### The Effects of Ethanol on AJ and TJ Protein and Gene Expression

**Figure 6.**
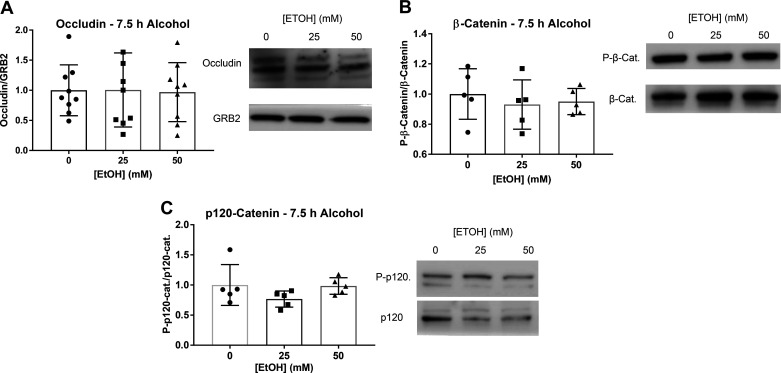
Changes in TJ and AJ protein expression after 7.5 h treatment with ethanol (0, 25, 50 mM). *A*: protein expression of occludin detection after 7.5 h ethanol. Claudin and VE-cadherin protein levels could not be detected. *B*: beta-catenin phosphorylation after 7.5 h ethanol. *C*: p120 phosphorylation after 7.5 h ethanol. Phosphorylation of p120 could not be detected after 30 min ethanol. Y-axis represent changes in phosphorylated protein/total protein. X-axis represents different concentrations of ethanol. GRB2 served as loading control. Circle, square, and triangle plots represent the groups receiving 0, 25, or 50 mM ethanol, respectively. A total of three independent experiments were performed for Fig. 6 data. Values are mean ± SE, *n* = 5–9; one-way ANOVA, Tukey’s *P* > 0.05. AJ, adherens junction; EtOH, ethanol; GRB2, growth factor receptor bound protein 2; tj, tight junction; VE-cadherin, vascular endothelial cadherin.

**Figure 7.**
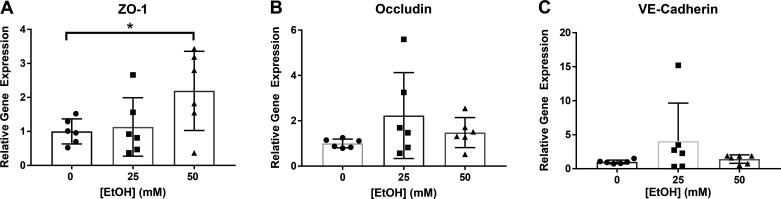
Relative gene expression of TJ and AJ proteins after 7.5 h ethanol (0, 25, 50 mM). *A*: ZO-1 gene expression. *B*: occludin gene expression. *C*: VE-cadherin gene expression. Y-axis represent relative changes in gene expression normalized to controls. X-axis represents different concentrations of ethanol. Circle, square, and triangle plots represent the groups receiving 0, 25, or 50 mM ethanol, respectively. A total of three independent experiments were performed for Fig. 7 data. Values are mean ± SE, *n* = 6; *t* test; *P < 0.05. AJ, adherens junction; EtOH, ethanol; TJ, tight junction; VE-cadherin, vascular endothelial cadherin; ZO, zonula occludens.

AJ and TJ protein and gene expression were measured by Western blot (WB) analysis and RT-qPCR. No significant changes were observed in levels of occludin protein expression after 7.5 h ethanol (Fig. 6*A*). Claudin-5 and ZO-1 could not be detected by WB. No significant changes in β-catenin or p120-catenin phosphorylation were observed after 7.5 h of ethanol (Fig. 6, *B* and *C*). ZO-1 gene expression significantly increased in cells treated with 50 mM ethanol (Fig. 7*A*). There were no significant changes in occludin or VE-cadherin gene expression (Fig. 7, *B* and *C*). Claudin-5 gene expression could not be detected. A few reasons claudin-5 was not detected in the LECs could be antibody concentration for Western blot and the RNA concentration that was transcribed was low for detection of claudin-5.

### Pharmacological Inhibition of p38 MAPK

**Figure 8.**
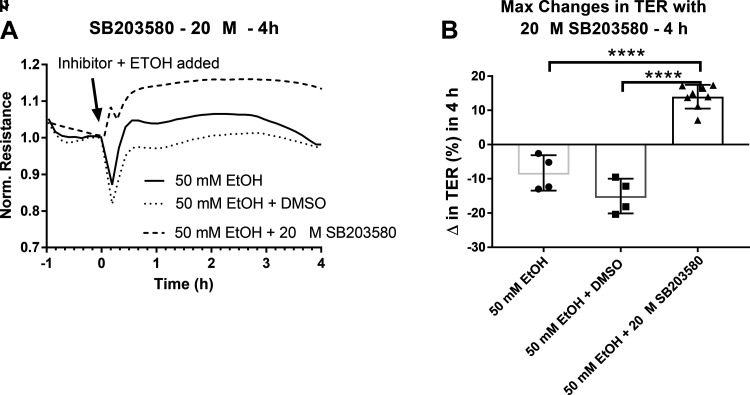
The effects of p38 inhibition on ethanol (50 mM)-induced decreases in TER. *A*: averaged tracings of each treatment group, Y-axis values is resistance measurements normalized to time point before treatment began (time zero), X-axis represents the entire time course. *B*: maximum changes (Δ) in TER with 20 µM p38 inhibitor + 50 mM EtOH. Circle, square, and triangle plots represent the groups receiving 50 mM ethanol, 50 mM ethanol + DMSO, and 50 mM ethanol + 20 µM SB203580, respectively. A total of three independent experiments were performed for Fig. 8 data. Values are mean ± SE; *n* = 4 for control and vehicle groups, *n* = 8 for inhibitor groups; one-way ANOVA, Tukey’s; ****P < 0.0001. EtOH, ethanol; TER, transendothelial resistance.

LECs exposed to 50 mM ethanol for 7.5 h showed significant increases in p38 MAPK activity as measured by WB. With these data and the known role of MAPK in the regulation of endothelial cell barrier function (30), we tested the role of p38 MAPK on ethanol-induced LEC barrier dysfunction. The p38 MAPK inhibitor SB203580 at 20 µM concentration was used to selectively inhibit p38 activity during exposure of LECs to ethanol at 50 mM concentrations. Previous studies with concentrations of this inhibitor ranging from 6 to 20 µM have shown successful inhibition of the p38 cascade while also preventing alcohol-induced increases in endothelial cell permeability (26). We determined the appropriate inhibitor concentration by diluting the inhibitor at various concentrations (1nM, 5 nM, 10 nM, 100 nM, 1 µM, 10 µM, and 20 µM) and measuring changes in LEC TER after treatment with 50 mM ethanol. We simultaneously monitored changes in TER during this treatment process to observe the effects of p38 inhibition on ethanol-induced permeability. 20 µM SB203580 prevented the ethanol-induced decrease in TER within the first four hours of ethanol treatment (Fig. 8*A*) and lead to an increase in TER during this time compared to control (50 mM ethanol) and vehicle (50 mM ethanol + DMSO) groups (Fig. 8*B*).

## DISCUSSION

Excessive alcohol exposure has been shown to cause increased collecting lymphatic vessel permeability (23), potentially causing both immune and metabolic changes observed after alcohol consumption (24, 25). However, the underlying mechanisms of alcohol-induced lymphatic hyperpermeability remain unclear. Previous in vitro studies using different endothelial cell types have shown that ethanol exposure causes increased permeability of endothelial monolayers via activation of MAPK signaling and disruption of TJ protein dynamics (26, 27, 30). The effects of ethanol on lymphatic endothelial cell barrier function and the signaling pathways involved in regulation of barrier function have yet to be studied. In this study, we demonstrate that ethanol disrupts the lymphatic endothelial barrier and increases activation of p38 MAPK. We further show that inhibition of p38 activity prevents ethanol-induced decreases in barrier function. While studies presented here were performed in dermal LECs, previous studies have characterized cell junctions in dermal LECs and have shown similarities in the distribution of TJs, AJs, and molecular markers in both dermal and visceral LECs, as well as initial and collecting lymphatic vessels (38, 45, 65). Therefore, we do not anticipate any significant differences between dermal and visceral LECs with respect to regional protein distribution.

Ethanol at 50- and 100-mM concentrations has been reported to increase permeability of endothelial cell monolayers as measured by transendothelial resistance (TER) (26). Using similar techniques, our studies show that when LECs are cultured with ethanol-supplemented medium, the TER of LEC monolayers significantly decreases over the course of 24 h compared to controls. This effect is concentration dependent, with 50 mM ethanol causing a greater and more sustained drop in TER compared to 25 mM ethanol. Additionally, after 24 h of treatment with ethanol, the TER of cells treated with 50 mM ethanol did not fully recover to baseline values measured before the addition of ethanol. These effects of higher concentrations of ethanol have been observed in various endothelial cell types (28, 29) and suggest that larger concentrations of ethanol cause a long-term change in barrier function of LECs.

While we observed that ethanol has no effect on cell viability after 7.5 h, decreases in cell viability seen in cells treated with 50 mM ethanol for 24 h suggests that ethanol may be decreasing viability over the course of the 24-h exposure. However, this disruption of LEC barrier integrity and viability is reversible in these alcohol-exposed cells once 50 mM ethanol is removed. Ethanol at 50 mM caused a greater change in TER that was sustained, or not recovered back to baseline resistance, after the first 24 h of ethanol exposure. Recovery of LEC monolayer resistance back to baseline 24 h after re-supplementation with normal medium suggests that the effects of higher concentrations of ethanol are more sustained but can be rescued back to baseline resistance. Previous studies have reported similar findings, showing that ethanol at approximately 40- and 80-mM concentrations decreased TER, and removal of ethanol led to reversal of this decrease (27). Although loss of cell viability could explain long-term increases in permeability at higher ethanol concentrations, further studies are needed to elucidate this relationship and the effects that higher ethanol concentrations have on cell health and cell division.

In addition to changes in LEC TER observed over the span of 24-h ethanol treatment, we also observed initial and acute changes in TER within the first 30 min of treatment. Although TER slightly increased in cells treated with control culture medium, 25- and 50-mM ethanol caused drops in TER that were observed within 30 min after addition of ethanol. Our previous studies in vivo have shown that animals administered alcohol acutely, in a binge, and chronically in the diet show increased lymphatic vessel permeability after 30 min of ethanol administration (23–25). Our results in vitro, therefore, suggest that changes in permeability measured by TER occurring within the first 30 min of ethanol supplementation are consistent with results seen in animal models of lymphatic vessel hyperpermeability. Additionally, similar to what we observed over 24 h of ethanol treatment, this early drop in TER after 30 min was both greater and more sustained in cells treated with higher concentrations of ethanol. Initial and sustained effects of ethanol on changes in permeability have been shown at time points as early as 10 min and maintained as late as 24 h (11, 27, 28, 57–59, 66–68). We observe an initial change in TER within the first 30 min of ethanol exposure followed by a sustained change in TER over the course of 24 h. Similarly, Xu et al. (27) have shown that human umbilical vein endothelial cells (HUVECs) treated with ethanol show an initial drop in TER that occurs within the first 10 min of ethanol treatment. This drop is sustained over the course of 24 h and is rescued after removal of ethanol (27), similar to what we observe in the present studies.

Although changes in TER are indicators of cell monolayer permeability, movement of molecules across a cell monolayer is another indicator of changes in barrier function. A transwell permeability assay was used to detect changes in LEC barrier function and migration of molecules across the cell barrier. We measured the amount of HRP that migrated across the LEC barrier at both 30 min and 7.5 h of treatment with ethanol to detect any initial changes in permeability, as well as the more sustained effects of changes in permeability. Although there was no significant difference between treatment groups in the amount of HRP migration after 30 min of alcohol, there was a significant increase in HRP migration in cells treated with 50 mM alcohol for 7.5 h.

In studies of endothelial barrier function, various permeability-inducing agents have been shown to decrease TER and increase monolayer migration in a concentration-dependent manner (27, 69–71). Although these agents show both instantaneous and sustained long-term changes in TER, permeability across cell monolayers measured as macromolecule migration was often not significantly increased until higher doses of permeability-inducing agents were used (58). Although our studies show initial and long-term decreases in TER at both 25 and 50 mM ethanol, significant increases in transwell permeability were only observed when cells were treated with 50 mM ethanol for 7.5 h. Although the observed decreases in TER of cells treated with 25 mM ethanol were significant compared to controls, this decrease was likely not large enough to allow a 44 kDa molecule to migrate across the cell monolayer. Similar results were seen at early time points of decreased TER. TER was significantly decreased in LECs exposed to ethanol within 30 min of ethanol treatment, but migration of HRP was not significantly different in these monolayers compared to controls after 30 min of ethanol exposure.

After 7.5 h of 50 mM ethanol treatment, we also found an increased amount of phosphorylated p38 MAPK compared to controls at 7.5 h of ethanol. However, ERK1/2 phosphorylation did not significantly increase after treatment with either 25 or 50 mM ethanol, and levels of JNK MAPK phosphorylation could not be detected. After 30 min of ethanol, there was no change in phosphorylation of p38 (data not showed).

Increased p38 MAPK phosphorylation has been shown in various endothelial cell types after treatment with barrier dysfunction-inducing agents. Additionally, inhibition of activated MAPK signaling pathways has been shown to prevent ethanol-induced increases in permeability, and inhibition of p38 phosphorylation specifically has led to amelioration of ethanol-induced endothelial cell monolayer permeability (26, 30). In addition to barrier dysfunction, disruption in TJ and AJ protein dynamics was also observed in these studies (26, 30, 72). Although p38 MAPK phosphorylation has been shown to be a major mechanism involved in regulation of TJ protein expression and cell monolayer permeability, the direct role of p38 in regulating ethanol-induced lymphatic endothelial cell permeability is unknown. We measured the relative changes in protein levels and gene expression of TJ and AJ proteins, but no changes were observed in levels of occludin after 7.5 h of ethanol treatment. We also did not observe any differences in phosphorylation of the VE-cadherin subunits β-catenin or p120-catenin after 7.5 h of ethanol treatment (Fig. 7). Gene expression measured by RT-qPCR showed a significant increase in ZO-1 gene expression of cells treated with 50 mM ethanol compared to controls. Claudin-5 gene expression could not be detected by RT-qPCR.

We hypothesized that ethanol activates LECs MAPK signaling and subsequently disrupts the LEC barrier via decreased TJ and/or AJ protein expression. Our results show that ethanol induced hyperpermeability in cells exposed to both 25 and 50 mM ethanol. This occurred at both 7.5 h and within the first 30 min of exposure. However, the only significant increase in p38-phosphorylation occurred in cell treated with 50 mM ethanol for 7.5 h. These findings suggests that changes in barrier function, occurring before or after 7.5 h, of cells exposed to 25 mM ethanol are not necessarily associated with changes in MAPK signaling. Although the results shown in Fig. 4 suggest that decreased cell viability may be responsible for increased permeability and p38 phosphorylation in cells exposed to 50 mM ethanol, other mechanisms could be involved in the discrepancies observed.

TJs and AJs are intercellular adhesion complexes that are involved in the barrier function of endothelial cells. They are involved in maintenance of cell polarity by limiting the movement of cells, proteins, and other molecules across the plasma membrane, and they help initiate and maintain cellular adhesion (32). Both junctional complexes are not only sites of attachment between endothelial cells, but they can also function as signaling structures that communicate and regulate various cell properties and functions, such as position, growth, and vascular homeostasis. In both junction types, adhesion is mediated by transmembrane proteins that promote homophilic interactions and form cell-cell connections along the cell border (34, 35, 45). Any change in junctional integrity, organization, protein expression, or function may have complex consequences that could compromise endothelial barrier function.

Because previous studies have shown that ethanol can cause decreases in junctional protein expression (30, 73), we expected TJ and AJ protein and gene expression to decrease after treatment with ethanol. However, we did not observe any significant decreases in protein levels or relative gene expression after treatment with ethanol. While increases in ZO-1 expression after treatment with 25 mM ethanol for 7.5 h may suggest a potential compensatory mechanism for ethanol-induced changes in permeability, similar findings have not been reported in the literature. As previously described, junctional protein expression does not have to be decreased for alterations in barrier function of endothelial cell monolayers to occur. Other mechanisms of permeability regulation may include: *1*) changes in localization of TJ and AJ proteins via rearrangement of the actin cytoskeleton (27, 74, 75), or *2*) alterations of vascular endothelial cadherin (VE-cadherin) dynamics and availability at the cell membrane via tyrosine phosphorylation (33, 76, 77).

Up- or downregulation of various lymphatic vessel genetic markers involved in LEC differentiation, including Lyve1 and Prox1, are also known to occur in various diseases related to the LS (43). These mechanisms could be implicated in alcohol-induced LEC hyperpermeability and could also explain rapid changes in LEC permeability that were observed within the first 30 min of ethanol exposure. Lastly, histamine, a known inducer of lymphatic permeability, has also been shown to be increased in alcohol-preferring rats, suggesting that this molecule could play a role in ethanol-induced hyperpermeability (78). The RhoA-Rho kinase (ROCK) pathway and cAMP both promote endothelial cell barrier function, and ROCK has been shown to serve as a mediator of histamine-induced barrier dysfunction in similar endothelial cell types (68). Further studies exploring the role of these potential mechanisms are needed to better understand the complete mechanism involved in time-varying changes of ethanol-induced LEC permeability.

### Inhibition of p38 MAPK Activity Prevents Decreases in Barrier Function

We assessed the potential role of p38 in ethanol induced barrier dysfunction by inhibiting p38 activity. We incubated LECs with the p38 MAPK inhibitor SB203580 at 20 µM for 1 h before the addition of ethanol and measured changes in TER over the course of 24 h. Inhibition of p38 activity significantly prevented ethanol-induced changes in TER within the first four hours of ethanol treatment. This finding suggests that p38 phosphorylation could be involved in the regulation of acute changes in barrier function during 50 mM ethanol treatment, and these results are consistent with previous findings in which p38 inhibition prevented early decreases in TER caused by ethanol, as well as other permeability-inducing agents (11, 26, 30, 57–59, 66, 67, 69, 79, 80). By-products of alcohol metabolism, including reactive oxygen species (ROS), have also be shown to induce endothelial cell barrier dysfunction and increase p38 MAPK activation (66, 67, 69, 79, 80). Because of these known inducers of p38 activity and increased permeability, by-products could be playing a role in alcohol-induced increases in p38 activity during long-term treatment with alcohol, but not during short term treatment. Future studies exploring the changes in p38 activity over an entire time course of 24 h could highlight the temporal changes in p38 activity that occur during ethanol treatment. This could also explain the significant increase in p38 activity we observed after 7.5 h of ethanol treatment but not after 30 min of ethanol treatment, suggesting that p38 activity could be increasing at earlier time points, but not enough to be significantly detected by Western blotting.

### Conclusions

We hypothesized that ethanol induces lymphatic endothelial cell permeability via disruption of lymphatic endothelial tight junctions through MAPK activation. Our results show that ethanol increases lymphatic endothelial cell (LEC) permeability and decreases transendothelial resistance (TER), correlating to changes in barrier function that are potentially linked to alterations in junctional protein dynamics. Ethanol did not significantly affect cell viability or cytotoxicity at 7.5 h. Moreover, loss of cell viability and decreases in TER after 24 h of 50 mM ethanol treatment recovered with control media supplementation. In addition to decreasing barrier function, 50 mM ethanol increased LEC levels of p38 MAPK phosphorylation compared to controls after 7.5 h of ethanol. p38 inhibition significantly prevented ethanol-induced decreases in barrier function within the first 4 h of ethanol treatment, but this was not replicated following longer periods of ethanol exposure. Previous in vivo studies showed that alcohol-fed animals have increased mesenteric lymphatic permeability and leakage of lymph into surrounding perilymphatic adipose tissue. The in vitro studies discussed here suggest similar effects of alcohol on LEC barrier function but fail to conclusively demonstrate that alcohol-induced activation of MAPK signaling alters junctional protein expression. There was no significant change in TJ or AJ protein expression or localization observed, mechanisms that have been previously shown to induce changes in barrier function. Therefore, future studies exploring various other signaling pathways and junctional proteins implicated in barrier function regulation would provide important insight into how ethanol is directly causing hyperpermeability of the lymphatic system in the presence of ethanol. Studying the effects of ethanol on the important structural components involved in this regulation would also strengthen our understanding of how endothelial cell anatomy may be altered during states of hyperpermeability.

## SUPPLEMENTAL DATA

Supplemental Fig. S1: https://figshare.com/s/ee8b8f15d2c51492ad05.

## GRANTS

This study was supported in part by National Institute on Alcohol Abuse and Alcoholism Grant K01 AA026640.

## DISCLOSURES

No conflicts of interest, financial or otherwise, are declared by the authors.

## AUTHOR CONTRIBUTIONS

F.M.S.-S. conceived and designed research; M.H. performed experiments; M.H. and F.M.S.-S. analyzed data; M.H., P.M., and F.M.S.-S. interpreted results of experiments; M.H. and F.M.S.-S. prepared figures; M.H. drafted manuscript; M.H., P.M., and F.M.S.-S. edited and revised manuscript; M.H., P.M., and F.M.S.-S. approved final version of manuscript.
